# Establishment of two oxaliplatin-resistant gallbladder cancer cell lines and comprehensive analysis of dysregulated genes

**DOI:** 10.1515/med-2023-0690

**Published:** 2023-09-27

**Authors:** Haijun Guo, Yunqing Zhi, Kaijing Wang, Na Li, Danlei Yu, Zhonghua Ji, Bo Chen

**Affiliations:** Department of Emergency Surgery, Shanghai University of Medicine & Health Sciences Affiliated Zhoupu Hospital, Shanghai 201318, China; Department of Assisted Reproductive Medicine, Shanghai First Maternity and Infant Hospital, Tongji University School of Medicine, Shanghai 201204, China; Department of Hepatobiliary Surgery, Shanghai East Hospital, Tongji University School of Medicine, Shanghai 200120, China; Department of Nursing, Shanghai East Hospital, Tongji University School of Medicine, Shanghai 200120, China; Department of Anesthesia, Shanghai East Hospital, Tongji University School of Medicine, 150 Jimo Road, Shanghai 200120, China; Department of Hepatobiliary Surgery, Shanghai East Hospital, Tongji University School of Medicine, 150 Jimo Road, Shanghai 200120, China

**Keywords:** oxaliplatin, gallbladder cancer, resistant cell lines, CSF2, gene expression profile

## Abstract

Acquired resistance to chemotherapeutic drugs in gallbladder cancer (GBC) results in therapy failure. This study is aimed to establish oxaliplatin (OXA)-resistant GBC cell lines and uncover their gene expression profiles. First, two OXA-resistant GBC cell lines (GBC-SD/OXA and SGC996/OXA) were established by gradually increasing the drug concentration, and the resistance index was 4–5. The two resistant cell lines showed slower proliferation and higher stemness, colony formation, and migration abilities. Epithelial mesenchymal transformation and increased levels of P-glycoprotein were also detected. Next RNA-sequence analysis identified 4,675 dysregulated genes (DGs) in resistant cells, and most of the 12 randomly selected DGs were verified to be consistent with the sequence results. Kyoto Encyclopedia of Genes and Genomes analysis indicated that several DGs were involved in resistance- and phenotype-related pathways, of which the activations of PD-L1 and ERK1/2 were both verified in resistant cell lines. In conclusion, this study is the first to report the gene expression profile of OXA-resistant GBC cells and provides a useful database for target development.

## 1 Introduction

Gallbladder cancer (GBC) is the most common malignant tumor of the biliary system, accounting for 0.6% of new cancer diagnoses worldwide [1]. Surgical resection is regarded as the most effective treatment for GBC; however, approximately 80% of patients have progressed to the advanced stage at the first diagnosis, thus not having the option of surgery. Therefore, the 5 year survival rate of patients with locally advanced or metastatic GBC is <5% [2]. To date, the combination of gemcitabine and platinum-based anticancer drugs remains the standard treatment for advanced or metastatic GBC [3]; however, the response rate and time are often unsatisfactory. The major reasons for this are the significantly higher molecular heterogeneity of GBC compared to other biliary system cancers, as well as the inevitable acquired resistance to chemotherapeutic drugs [4].

Next-generation sequencing technology is an effective tool for identifying new targetable alterations for precision medicine. A series of oncogenic mutations have been found in patients with GBC, including ERBB2 (HER2) amplification, MEK mutation, and BRAF mutation [5,6], providing new options for the treatment of GBC. However, these attempts at targeted or combination therapies are still less desirable, especially in resistant patients. For instance, a clinical trial indicated that blockage of HER2 was beneficial in some GBC patients with HER2 alteration, whereas no response was observed in others [7]. Dabrafenib (a BRAF inhibitor) combined with trametinib (an MEK inhibitor) has a 51% objective response rate in biliary system cancers [8]; however, trametinib has no activity in advanced GBC resistant to gemcitabine-based chemotherapy [9]. The limited effect of these targeted therapies indicates that other compensatory pathways must be activated in cancer, and the existing potential targets are insufficient for the effective treatment of advanced or resistant GBC.

Compared to other cancers, the underlying mechanisms for GBC resistance have been less investigated, and the major reasons are the rarity of clinical cases/samples as well as the lack of resistant cell lines. Most clinical trials related to GBC are included in the studies of biliary system cancers [7,8,9], and trials focusing only on GBC are numbered. Therefore, the establishment of resistant cell lines is of great significance for identifying more potential targets. Oxaliplatin (OXA) is one of the most commonly used platinum compounds in GBC [10,11], and acquired resistance to OXA is often neglected compared to more widely used gemcitabine. The present study is aimed to establish two OXA-resistant GBC cell lines and detect phenotypic alterations in the resistant cells. RNA-sequence (RNA-seq) and bioinformatics analyses were performed to identify dysregulated genes (DGs) in resistant cells and classify the biological roles of DGs. Several DGs and pathways were selected for verification. We aimed to establish the gene expression profile of OXA-resistant cells, thereby providing a solid foundation for the combined targeted therapy of GBC.

## 2 Materials and methods

### 2.1 Cells and cell culture

The human GBC cell line GBC-SD was purchased from the Cell Bank of the Chinese Academy of Sciences (Shanghai, China) and SGC996 was purchased from the American Type Culture Collection (ATCC, Manassas, VA, USA). The two sensitive cell lines were cultured in Dulbecco’s modified eagle’s medium (DMEM; BBI Life Science, Shanghai, China) supplemented with 10% fetal bovine serum (FBS; BI, Israel) and 1% penicillin/streptomycin (BBI Life Science). Cells were maintained in an incubator with 5% CO_2_ at 37℃.

### 2.2 Establishment of OXA-resistant GBC-SD and SGC996 cell lines

The sensitive cell lines GBC-SD and SGC996 were seeded in six-well plates, and resistant cells were induced by gradually increasing the drug concentration. The process can be divided into four stages. Stage I: The initial concentration of OXA (Selleck, Shanghai, China) was 0.05 μM and the passage cycle was controlled at 2–3 days at a seeding density of 2 × 10^5^ cells/well. This stage lasted for 2–3 weeks until the cells were fully adapted to the initial dose. Stage II: The dose was gradually increased until the cells could survive and proliferate in medium containing 5 μM OXA, and this stage lasted for 10–12 weeks. The passage cycle was controlled for 4–5 days at a seeding density of 3 × 10^5^ cells/well. Stage III: The achieved cells were preserved in liquid nitrogen for a month and then recovered and cultured with 5 μM OXA in every passage for an additional month. The passage cycle and seeding density were similar to those of stage II. In stage III, cells underwent an additional cycle of cryopreservation and normal growth with 5 μM OXA to obtain stable OXA-resistant cell lines, referred to as GBC-SD/OXA and SGC996/OXA. Stage IV: OXA (1 μM) was used as the common dose in long-term cryopreservation and passage culture. Cell morphology was observed and photographed using a microscope. Only the resistant cells at stage IV were used for the following assays, including RNA-seq analysis.

### 2.3 Drug-sensitivity assay

Two sensitive and two resistant cell lines were seeded at 5 × 10^3^ cells/well in 96-well plates, and different concentrations of OXA (0, 0.01, 0.1, 1, 10, and 100 µM) were added to the wells. Each concentration was used in four replicate wells. After 72 h of culture, the absorbance of each well was measured at 490 nm using an 2,5-diphenyl-2*H*-tetrazolium bromide (MTT) kit (CT01; Millipore), following the manufacturer’s protocols. The survival rate (%) was calculated according to the following equation: Cell viability (%) = OD of the experimental group/OD of the control group × 100. The drug concentration–viability curve and half-inhibitory concentration (IC_50_) were calculated using GraphPad Prism 5.0 (GraphPad Software, La Jolla, CA, USA). The drug resistance index (RI) was calculated according to the following equation: RI = IC_50_ of resistant cell line/IC_50_ of sensitive cell line.

### 2.4 Proliferation assay

The four cell lines were seeded at a density of 3 × 10^3^ cells/well in 96-well plates. The viability of the cells was measured using the MTT kit daily from days 1–7. Four replicate wells were used for each experiment. The proliferation rate was calculated using the equation: Proliferation rate (%) = OD of day *n*/OD of day 1, (*n* = 1–7).

### 2.5 Migration assay

The cells were collected and washed twice with FBS-free DMEM. An 8 μm pore polycarbonate membrane Boyden chamber insert in a Transwell apparatus (Millipore, MA, USA) was used to detect cell motility. In brief, 4 × 10^4^ cells resuspended in 0.2 mL FBS-free DMEM were seeded in the upper chamber, and 0.6 mL of the complete DMEM was added to the lower chamber. After incubation for 24 h, the cells on the upper surface were removed and the migrated cells on the lower surface were stained with 0.1% crystal violet for 10 min. Finally, the number of migrated cells was determined by counting the stained cells in three randomly selected fields using a microscope. The assay for each cell line was performed in triplicates.

### 2.6 Western blot

Total protein from the four cell lines was lysed using RIPA buffer (Beyotime, Shanghai, China) and quantified using a Bradford protein assay kit (MultiSciences, Hangzhou, China), following the manufacturer’s instructions. Next 10% sodium dodecyl-sulfate polyacrylamide gel electrophoresis was performed, and 10 μg of protein was added to the lanes. After electrophoresis was completed, separated proteins distributed in the gels were transferred into the 0.22 μm PVDF membranes. Subsequently, the membranes were incubated with 5% non-fat milk for 2 h and washed thrice with Tris-buffered saline with 0.1% Tween® 20 (TBST). The membranes were then cut into several pieces following the molecular weight of the target proteins, and incubated with the corresponding primary antibody overnight at 4℃, followed by the incubation with the secondary antibody at room temperature for an additional 2 h. Finally, the bands were incubated with the enhancement buffer following the steps of the ECL kit (Beyotime), and the signals were captured using an imaging system. The primary antibodies, E-cadherin, vimentin, N-cadherin, P-glycoprotein (P-gp), ERK1/2, p-ERK1/2, PD-L1, and the control, GAPDH, were purchased from ABclonal Biotech (Wuhan, China) and diluted 1,000-fold before incubation. The secondary antibody (1:2,000) was purchased from Cell Signaling Technology (Danvers, MA, USA). The gray values of the bands were analyzed using ImageJ software (National Institutes of Health, MD, USA).

### 2.7 Colony formation assay

For each cell line, 2 mL of complete medium containing 400 cells was added to a six-well plate. After 3 days of culture, 1 μM OXA was added to each well and the cells were cultured with OXA for an additional 11 days. The medium was changed every 4 days. Finally, the supernatant was removed and the cells were stained with 0.1% crystal violet for 20 min. Colonies containing >30 cells were counted and the assay was repeated thrice.

### 2.8 Soft agar colony formation assay

First, 1.5 and 0.8% agarose solutions were prepared in phosphate-buffered saline and subjected to high-pressure sterilization. Next a 1.5% agarose solution was mixed with an equal volume of medium and pre-coated in a 6-well plate. After the agarose solution solidified, 0.8% agarose solution mixed with an equal volume of medium containing 1,200 cells was seeded into each well. Colony formation lasted for 14 days, and 200 μL of medium was added to each well every 2 days.

### 2.9 RNA-seq analysis

Total RNA was collected from the four cell lines using TRIzol reagent (Invitrogen, Carlsbad, CA, USA) and extracted using an RNeasy mini kit (Qiagen, Germany). RNA quality and concentration were determined using a NanoDrop ND-1000 spectrophotometer (NanoDrop Technologies, Montchanin, DE, USA) and assessed using 1% gel electrophoresis. Next the four qualified RNA samples were sent to Sinotech Genomics Co., Ltd (Shanghai, China), for cDNA library construction and RNA sequencing. Gene abundance was expressed as fragments per kilobase of exon per million reads mapped (FPKM). StringTie software was used to count the fragments within each gene, and the TMM algorithm was used for normalization. The DGs between the sensitive and resistant groups were screened under the threshold of |log2 (fold change [FC])| > 1.

### 2.10 Quantitative polymerase chain reaction (qPCR)

Total RNAs were extracted from the four cell lines using a Universal RNA Extraction Kit (TAKARA, Dalian, China), following the manufacturer’s protocol, and then quantified using the NanoDrop ND-1000 spectrophotometer (NanoDrop Technologies). Next 1 μg of RNA was reverse-transcribed into cDNA using a PrimeScript RT reagent Kit (TaKaRa), according to the manufacturer’s instructions. For quantification of the target genes, the reaction system (20 μL) containing 2 μL of template, 10 μL of TaqMan Universal PCR Master Mix (Applied Biosystems), 1 μL of forward and reverse primers, and 7 μL of water was prepared and subjected to a Bio-Rad CFX96 Thermal Cycler (Bio-Rad, Hercules, USA). The thermal cycling conditions were as follows: 95℃ for 5 min, followed by 40 cycles at 95℃ for 15 s, and 60℃ for 60 s. Relative expression levels of mRNAs were calculated using the 2^−ΔΔCT^ method, and GAPDH was used as the internal control. The sequences of the primers listed in Table 1 were synthesized by Sangon Biotech (Shanghai, China).

**Table 1:** Primer sequences used for qPCR

Genes	Sequences (5′−3′)	
CCL20	Forward	TGCTGTACCAAGAGTTTGCTC
	Reverse	CGCACACAGACAACTTTTTCTTT
S1PR1	Forward	TCTGCTGGCAAATTCAAGCGA
	Reverse	GTTGTCCCCTTCGTCTTTCTG
CSF2	Forward	TCCTGAACCTGAGTAGAGACAC
	Reverse	TGCTGCTTGTAGTGGCTGG
TFF3	Forward	CCAAGCAAACAATCCAGAGCA
	Reverse	GCTCAGGACTCGCTTCATGG
FOXS1	Forward	AGTGGCATCTACCGCTACATC
	Reverse	CACCTTGACAAAGCACTCGT
FGG	Forward	AGACACGGTGCAAATCCATGA
	Reverse	GCCCGCTCTGTTTAGCTCC
CACNA1C	Forward	GAAGCGGCAGCAATATGGGA
	Reverse	TTGGTGGCGTTGGAATCATCT
EVI2A	Forward	CCCACGGACATGGAACACA
	Reverse	CACAGACGGGTATAGTTTGCTT
DLX1	Forward	TGCCAGAAAGTCTCAACAGCC
	Reverse	CGAGTGTAAACAGTGCATGGA
GNG4	Forward	GAGGGCATGTCTAATAACAGCAC
	Reverse	AGACCTTGACCCTGTCCATAC
MED29	Forward	ACGCAGTCGTAACGCACTT
	Reverse	AGCCGAACTAGGACCCGATAC
CXCL8	Forward	TTTTGCCAAGGAGTGCTAAAGA
	Reverse	AACCCTCTGCACCCAGTTTTC
GAPDH	Forward	GGAGCGAGATCCCTCCAAAAT
	Reverse	GGCTGTTGTCATACTTCTCATGG

### 2.11 Bioinformatical analysis

Gene Ontology (GO) analysis for biological processes, cellular components, and molecular function and Kyoto Encyclopedia of Genes and Genomes (KEGG) pathway analysis (http://www.genome.ad.jp/kegg) were performed to clarify the potential biological roles of DGs using the enrich R package. Enrichment analysis was conducted to list the top 30 GO terms and KEGG pathways.

### 2.12 Statistical analysis

Statistical analysis was performed using SPSS software (version 20.0; Chicago, IL, USA), and GraphPad Prism 5.0 (La Jolla, CA, USA) was used for data presentation. Statistical significance was tested using the Student’s *t*-test. *p* < 0.05 was considered statistically significant.

## 3 Results

### 3.1 Drug-sensitivity detection of OXA-resistant cell lines

**Figure 1:**
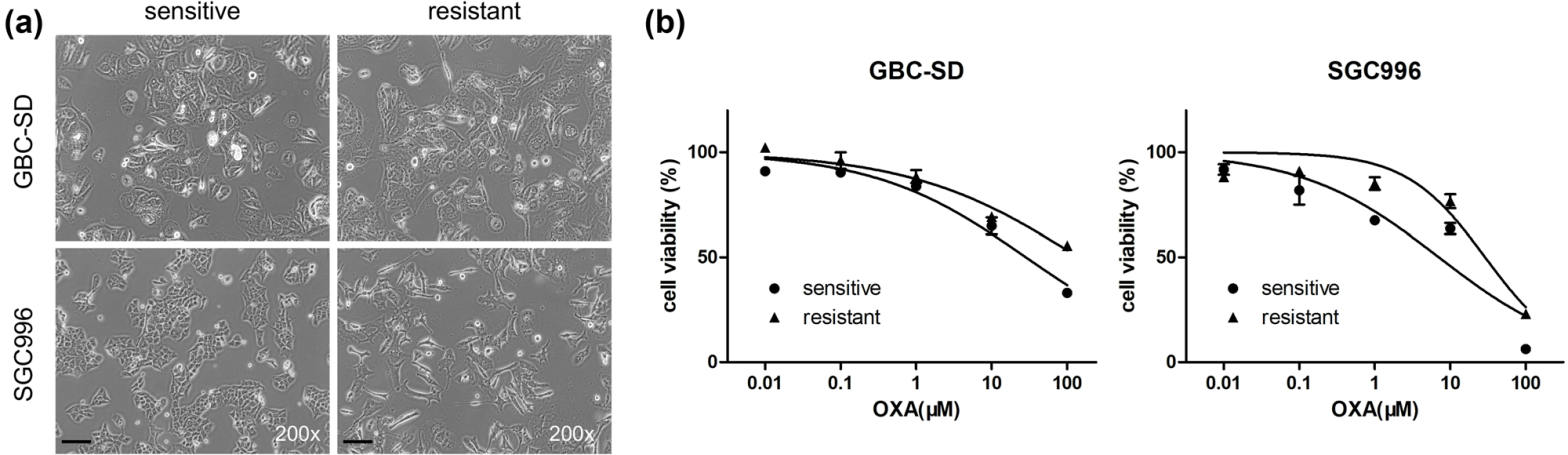
Drug-sensitivity detection of OXA-resistant cell lines. (a) Morphological character of resistant cell lines. Scale bar: 100 μM, magnification: 200×. (b) Cells were incubated with different concentrations of OXA and the MTT assay was used to detect drug-sensitivity.

The morphology of GBC-SD and GBC-SD/OXA was fusiform, and the resistant cells were larger than the parental cells (Figure 1a). Sensitive SGC996 cells were small and polygonal, whereas the resistant cells became significant spindles, and some of them had several pseudopodia (Figure 1a). In addition, the two sensitive cell lines tended to aggregate, whereas the resistant cells were both more dispersed (Figure 1a), which may be related to the alteration of motility.

An MTT assay was used to detect the drug sensitivity of the four cell lines. As shown in Figure 1b, GBC-SD and GBC-SD/OXA cells were similar in response to low and medium doses of OXA (0.01–10 μM), and GBC-SD/OXA showed a significantly higher resistance to 100 μM OXA. The IC_50_ values of GBC-SD and GBC-SD/OXA were 28.49 and 141.10 μM, respectively, and the RI value was 4.95. SGC996 cells were more sensitive to OXA than SGC996/OXA at in the dose of 1–100 μM. The IC_50_ value of SGC996/OXA cells was 29.02 μM, which was approximately 4-folds that of the sensitive cells (7.08 μM). These results confirm that the OXA-resistant cell lines GBC-SD/OXA and SGC996/OXA were successfully established.

### 3.2 Phenotype alterations of OXA-resistant cells

**Figure 2:**
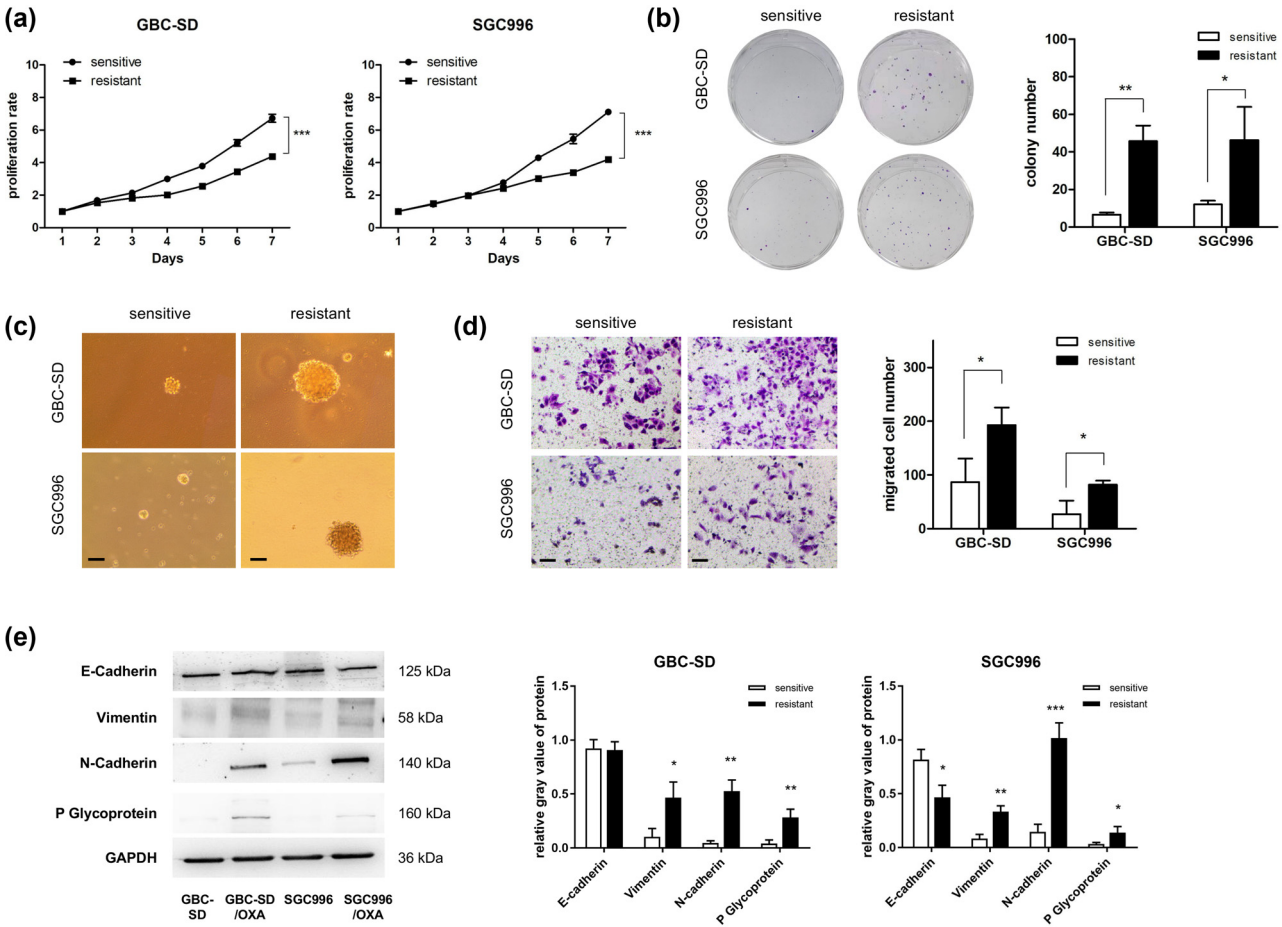
Phenotype detection of OXA-resistant cells. (a) Cells were cultured for 1–7 days, and the MTT assay was used to detect the proliferation rate daily. (b) Cells were cultured for 3 days and incubated for an additional 11 days in the presence of 1 μM OXA to evaluate colony formation ability. (c) The soft agar colony formation assay was performed to detect cell sphere formation ability. Scale bar: 100 μM. (d) Transwell assays were performed to detect the migration ability. Scale bar: 100 μM. (e) Western blotting was used to analyze EMT and P-glycoprotein markers.

As shown in Figure 2a, the proliferation rates of GBC-SD and its resistant cells were similar in the first 3 days of the study, and GBC-SD/OXA exhibited a slower growth rate than the sensitive cells in the last 3 days. Similar proliferation characteristics were observed in the SGC996/OXA cells (Figure 2a). Clone formation assay results indicated that resistant cells showed a stronger cloning potency than sensitive cells (Figure 2b) in the presence of 1 μM OXA, suggesting better tolerance of the resistant cells. Furthermore, soft agar colony formation assay results also indicated a higher three-dimensional cell sphere formation ability of the two resistant cell lines (Figure 2d), which indicated better stemness after cells were resistant to OXA. In addition, the migration abilities of the two resistant cell lines were significantly enhanced by the Transwell assay (Figure 2d). Western blotting results showed that vimentin and N-cadherin protein expression was both significantly increased after cells were resistant to OXA (Figure 2e). There was no significant change in E-cadherin expression in GBC-SD/OXA and GBC-SD cells, whereas its level was decreased in SGC996/OXA compared to that in the sensitive cells (Figure 2e). Overall, the resistant cells underwent epithelial mesenchymal transformation (EMT), consistent with their enhanced migration ability. One of the classic drug-resistant proteins, P-gp, was also detected, and the results showed a significant increase in the protein levels in the resistant cells (Figure 2e).

### 3.3 RNA-seq analysis of gene expression profile of OXA-resistant cell lines

**Figure 3:**
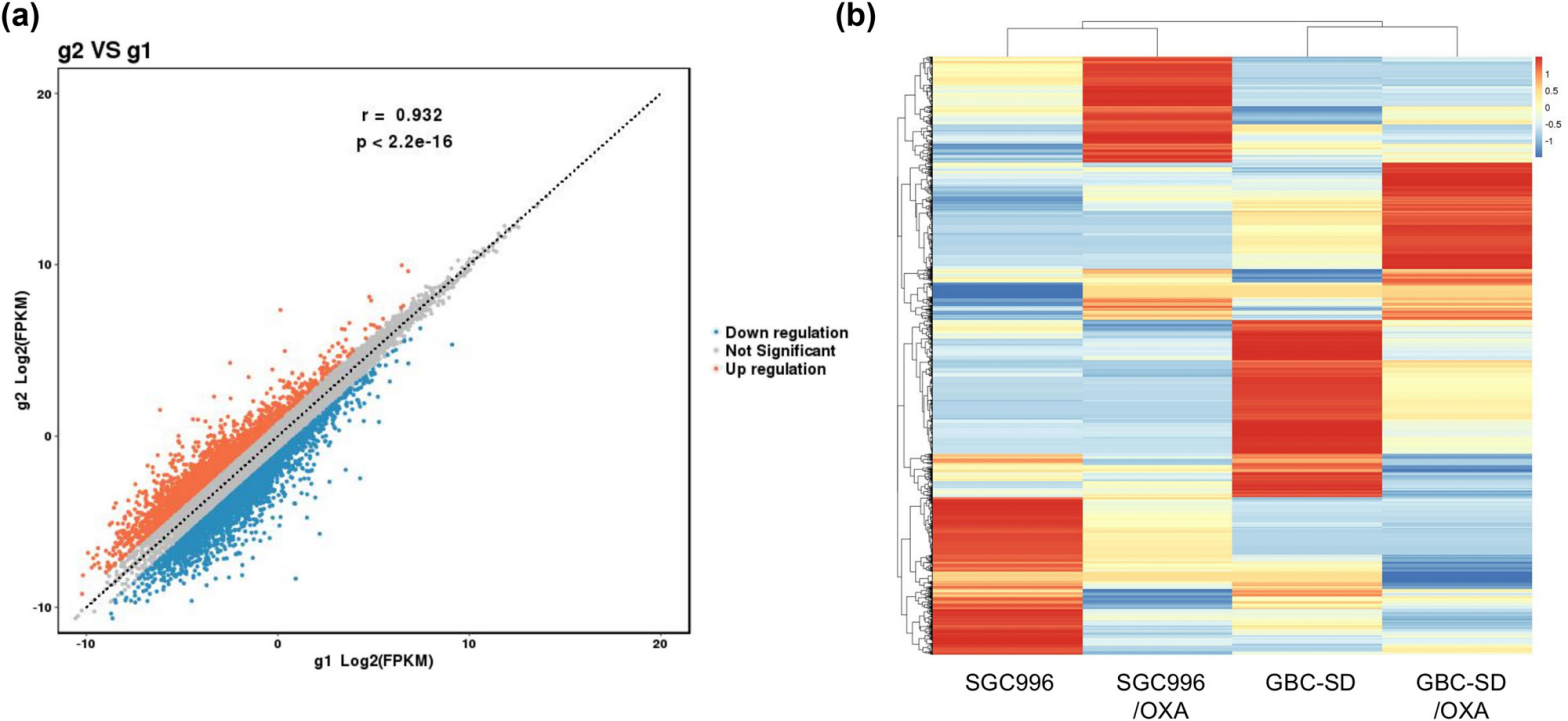
RNA-seq analysis of gene expression profile of OXA-resistant cell lines. (a) Scatter plot of DGs in the resistant groups. G2: resistant group, G1: sensitive group. (b) Heat map summarizing the expression of the DGs in each cell line. The color (red-blue) indicates that the FPKM values vary from +∞ to −∞. Each line represents a gene and each column represents a sample or cell line.

A total of 4,675 DGs were screened under the threshold of |log2[fold change (FC)]| > 1, including 2,060 upregulated and 2,615 downregulated genes (Figure 3a). The heatmap summarizes the gene expression of all DGs in the four samples, and the colors in each line indicate the FPKM values varying from +∞ to −∞ of the gene in each sample (Figure 3b). The gene expression files of the two different sensitive cells varied significantly, and several of them showed opposite FPKM values (positive and negative numbers). This was one major reason for the p-value not being considered in the screening of DGs. Interestingly, there was a significant difference in the FPKM values between the sensitive and resistant cell lines. The calculation of the final FC value was based on the mean FPKM value of the two sensitive/resistant samples in the same group; thus, thousands of genes were screened although there was no significant alteration in some genes (for example, similar watchet blue of FPKM values) between each pair of the sensitive and resistant cell lines. The DGs identified using RNA-seq provided several candidates for the study of resistance mechanisms, which also required further validation. The FC values of all DGs are listed in Table A1.

### 3.4 Validation of the DGs by qPCR

**Figure 4:**
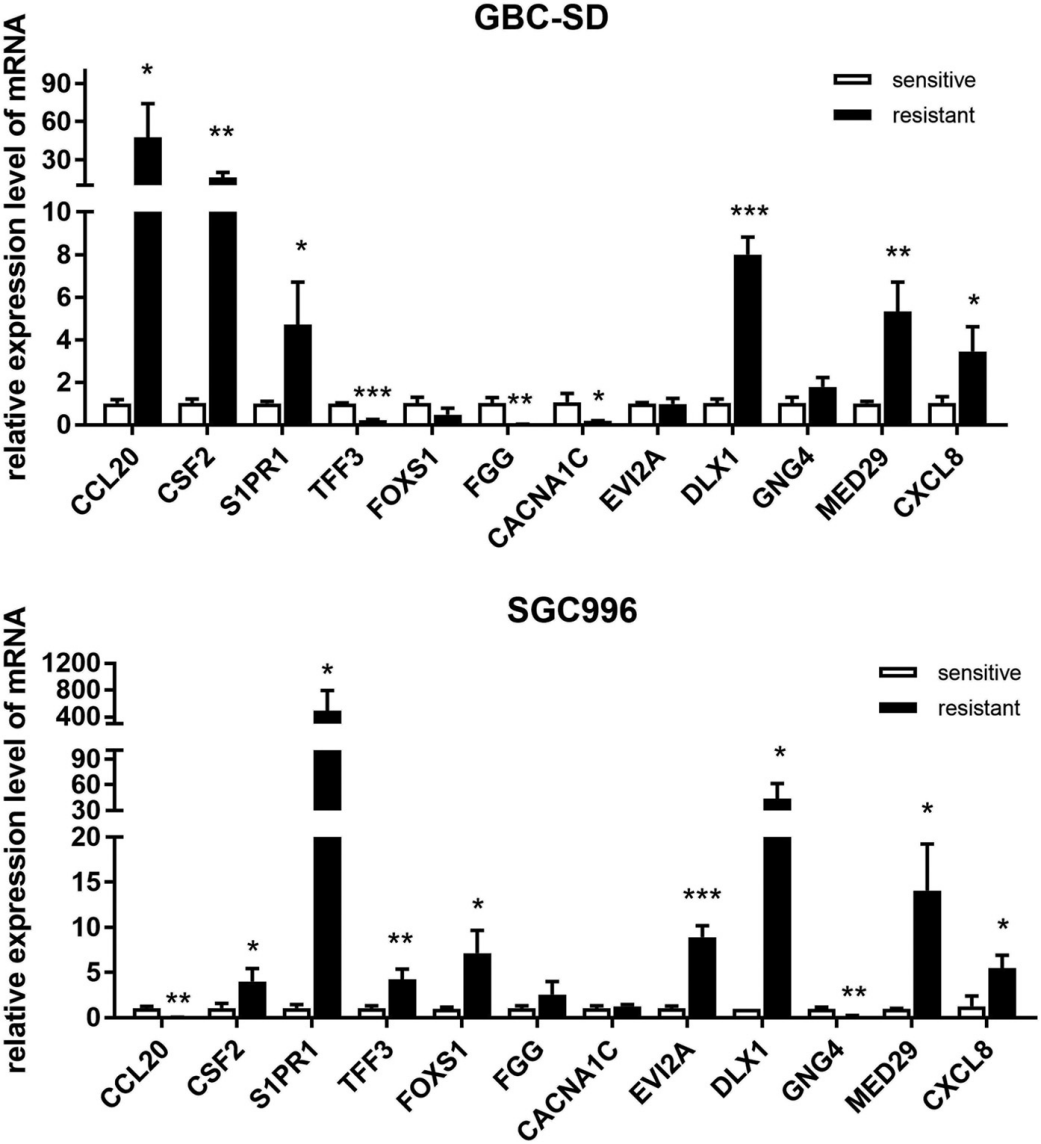
qPCR validation of the mRNA levels of 12 DGs in resistant cell lines.

Because the alteration trend of FPKM values of some DGs in each pair of cell lines was inconsistent, 12 genes were randomly selected from the top 300 DGs used for qPCR validation. The FPKM and FC values are presented in Table 2. 50% of the FPKM values in SGC996 cells and its resistant cells were 0, and the calculated log2FC value was predominantly contributed by GBC-SD and its resistant cells. The qPCR validation results indicated that the expression levels of most of the selected DGs were significantly changed in each resistant cell line (Figure 4). Among the 12 randomly selected genes, the expression changes of the 9 genes, accounting for 75%, in GBC-SD/OXA were consistent with the RNA-seq results. For SGC996/OXA, the alteration trends of five genes (CSF2, S1PR1, DLX1, MED29, and CXCL8) were conforming to the final log2FC values of RNA-seq (Figure 4, Table 2). In addition, the mRNA levels of above five genes were also both significantly enhanced in the two resistant cell lines (Figure 4). Overall, the RNA-seq results were reliable and satisfactory through the validation of random DGs, although some FPKM values were zero.

**Table 2:** Fold changes of 12 randomly selected genes among top 300

	Gene ID	Gene name	FPKM				FPKM (mean value)		log2FC	log2FC abs	FC abs
			GBC-SD/OXA	SGC996/OXA	GBC-SD	SGC996	G2	G1			
1	ENSG00000115009	CCL20	21.866	0.000	0.626	0.069	10.933	0.347	4.98	4.98	31.48
2	ENSG00000164400	CSF2	3.819	0.000	0.256	0.000	1.910	0.128	3.90	3.90	14.90
3	ENSG00000170989	S1PR1	0.000	0.110	0.000	0.005	0.055	0.003	4.38	4.38	20.89
4	ENSG00000160180	TFF3	0.032	0.000	1.490	0.000	0.016	0.745	−5.53	5.53	46.21
5	ENSG00000179772	FOXS1	0.054	0.000	2.121	0.000	0.027	1.061	−5.30	5.30	39.47
6	ENSG00000171557	FGG	0.074	0.000	5.745	0.000	0.037	2.872	−6.28	6.28	77.71
7	ENSG00000151067	CACNA1C	0.095	0.022	1.491	0.000	0.058	0.746	−3.68	3.68	12.79
8	ENSG00000126860	EVI2A	0.015	0.000	0.158	0.000	0.007	0.079	−3.40	3.40	10.59
9	ENSG00000144355	DLX1	0.044	0.251	0.021	0.008	0.148	0.014	3.35	3.35	10.21
10	ENSG00000168243	GNG4	0.000	0.058	0.000	0.006	0.029	0.003	3.25	3.25	9.50
11	ENSG00000063322	MED29	7.026	6.690	1.018	0.540	6.858	0.779	3.14	3.14	8.81
12	ENSG00000169429	CXCL8	471.258	5.653	55.490	3.152	238.456	29.321	3.02	3.02	8.13

G2: resistant group; G1: sensitive group; FC: fold change.

### 3.5 Bioinformatical analyses of DGs and western blot validation of some pathways

**Figure 5:**
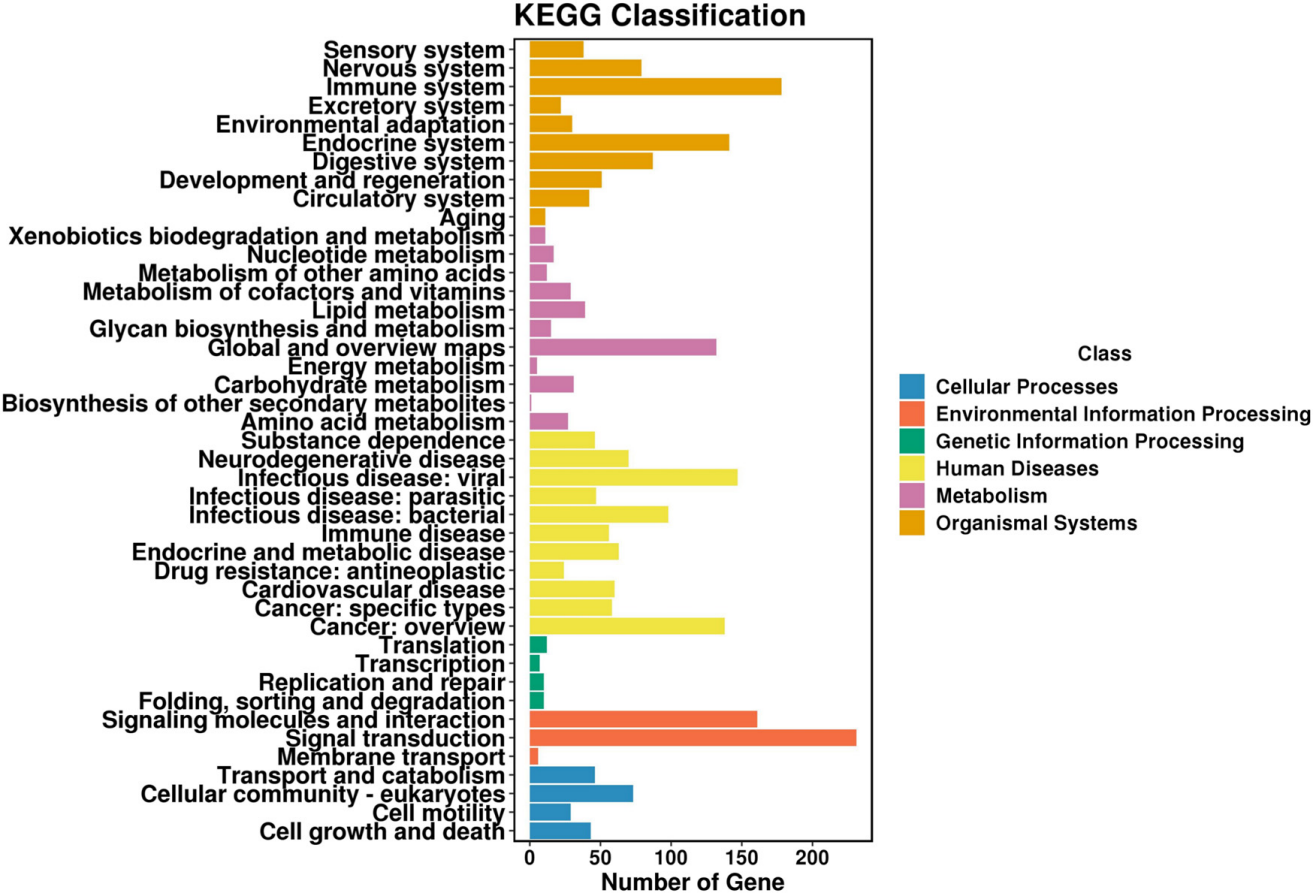
KEGG classification analysis of distribution of DGs in biological pathways.

**Figure A1:**
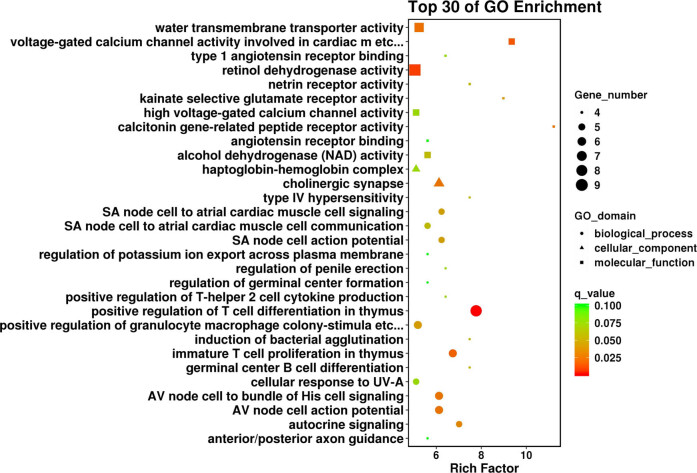
Top 30 terms in GO enrichment analysis.

**Figure A2:**
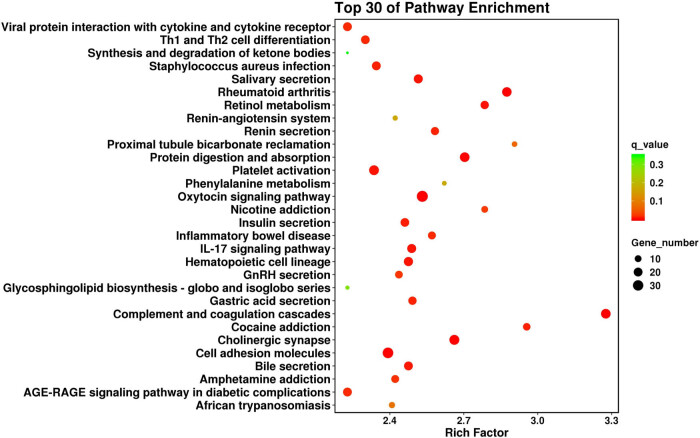
Top 30 pathways in KEGG enrichment analysis.

GO enrichment analysis indicated that DGs were enriched in several receptor/transporter activity-related molecular functions, including calcitonin gene-related peptide receptors, kainate selective glutamate receptors, and water transmembrane transporters (Figure A1). Some biological processes were also included in the top 30 GO terms, such as AV node cell-to-bundle of His cell signaling and SA node cell-to-atrial cardiac muscle cell communication (Figure A1). These results show that DGs are involved in the regulation of the activity of some receptors/transporters or cell signaling transduction, which may be related to chemical tolerance. KEGG classification results shown in Figure 5 indicate that >100 DGs were classified into pathways of cancer: overview, signal transduction, and immune system. Approximately 50 DGs were found to be directly involved in various cellular processes (Figure 5). Enrichment analysis indicated that the DGs were enriched in the metabolism of some molecules, including retinol, phenylalanine, and ketone bodies (Figure A2).

**Figure 6:**
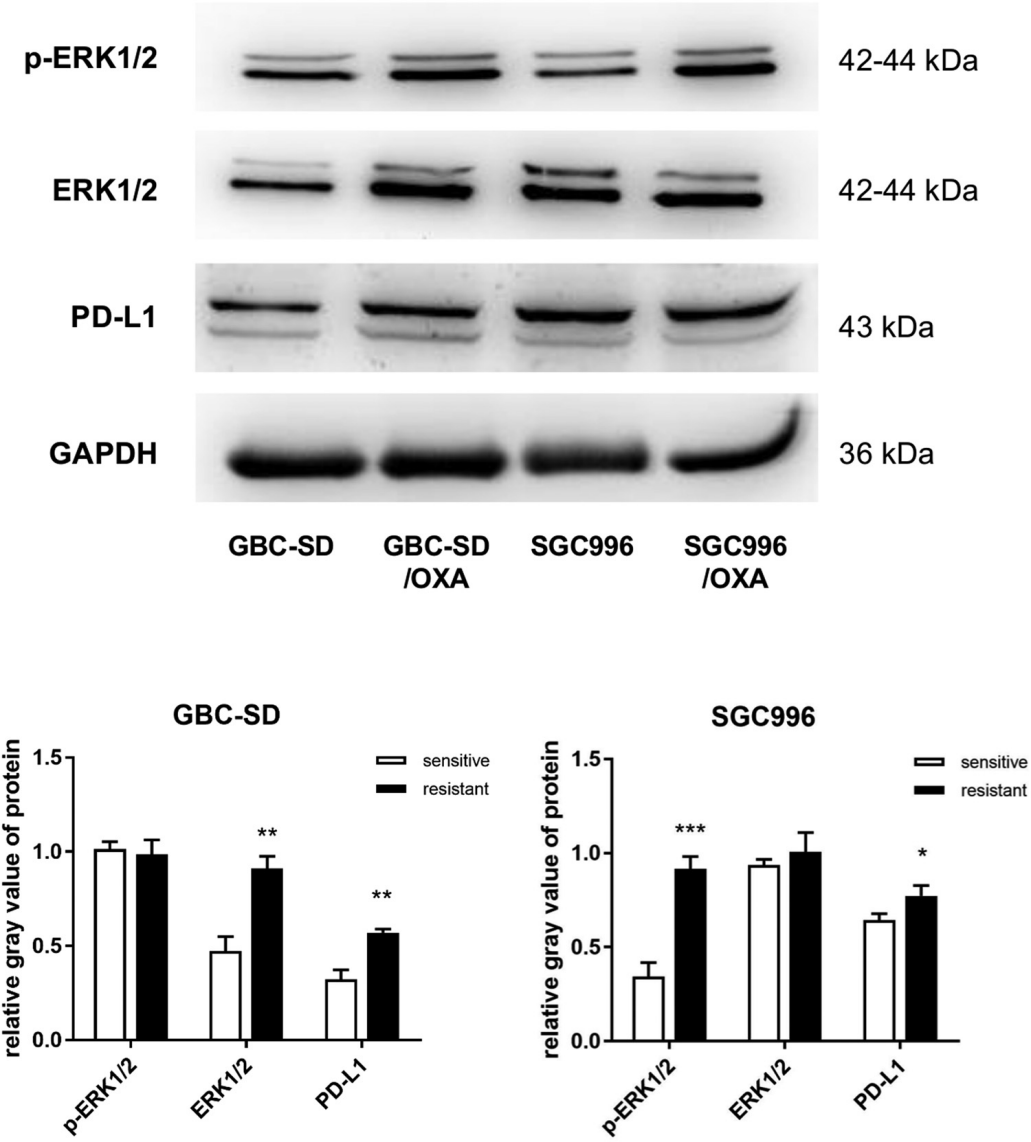
Western blot detection of the expression of PD-L1 and ERK1/2 pathways.

Resistant cells showed decreased proliferation and increased migration potency. Several relevant pathways were extracted from the KEGG classification results and are listed in Table 3. In the antineoplastic pathway of drug resistance, two sub-pathways were included: platinum drug resistance and EGFR tyrosine kinase inhibitor (TKI) resistance. Twelve DGs are involved in PD-L1 expression and the PD-1 checkpoint pathway in cancer, including CD247, also known as PD-L1. Notably, 27 DGs were classified into the transcriptional misregulation pathway in cancer. Some of them encode histones (H3C3, H3-4, H3C1, and H3C7) and some are inflammatory molecules (IL6 and CXCL8). These DGs are able to regulate transcription activity in various ways, which may be closely associated with the phenotypic alteration of the resistant cells. Some growth-related sub-pathways were also included, including the p53 pathway, the cell cycle, and oocyte meiosis. CDKN2A and CDKN1A are important regulators of cell cycle progression, and may be involved in slower proliferation. Three signaling pathways were randomly selected, and the MAPK pathway had the highest number of DGs (Table 3). To verify the relationship between the pathways and resistant cells, two pathways (MAPK and PD-L1) were selected for western blot analysis. The results in Figure 6 show that the expression of PD-L1 was significantly increased in resistant cells. The expression of p-ERK1/2 in SGC996/OXA was also significantly enhanced, whereas its level did not change in GBC-SD/OXA cells. Interestingly, the expression level of ERK1/2 in GBC-SD/OXA was significantly increased; therefore, the actual level of p-ERK1/2, but not the relative level, was significantly higher than that of the sensitive cells. The increased level of PD-L1 and activated ERK pathway may be potential mechanisms for acquired resistance.

**Table 3:** Several KEGG pathways and relevant DGs

Pathway_3	Pathway_3_num	All_diff_gene_list	Pathway_2	Pathway_2_num	Pathway_1	Pathway_1_num
Platinum drug resistance	5	CDKN2A, ATM, AKT3, CDKN1A, GSTA4	Drug resistance: antineoplastic	24	Human Diseases	389
EGFR TKI resistance	7	IL6, GAS6, PRKCG, AKT3, GRB2, PDGFRA, JAK2				
PD-L1 expression and PD-1 checkpoint pathway in cancer	12	TICAM2, IFNGR1, BATF2, MAP2K6, JUN, PRKCQ, CD247, LAT, AKT3, JAK2, RASGRP1, NFATC2	Cancer: overview	138		389
Transcriptional misregulation in cancer	27	LMO2, WNT16, GZMB, CEBPE, H3C3, IL6, NFKBIZ, MLLT3, MAF, H3-4, H3C1, CSF2, ATM, PROM1, SPI1, H3C7, MEF2C, MMP3, CXCL8, BCL2A1, CSF1R, CDKN1A, ITGB7, BCL11B, CEBPA, PLAT, PAX3				
Regulation of actin cytoskeleton	29	FGFR1, INSRR, CHRM3, ARHGEF6, ITGA10, FGFR4, ITGA11, BDKRB2, FGF9, KNG1, FGF3, CHRM5, ITGB6, SCIN, ITGB3, MYLK, MYH11, PAK3, MYLK3, FGF18, MYH10, ITGAX, PDGFRA, ITGAD, ITGB7, MYL9, MYLK2, FGF17, CHRM1	Cell motility	29	Cellular Processes	158
p53 signaling pathway	9	SESN3, CDKN2A, SERPINB5, SESN2, ATM, MDM4, CDKN1A, SERPINE1, ADGRB1	Cell growth and death	43		
Cell cycle	4	CDKN2A, ATM, CDKN1A, WEE2				
Oocyte meiosis	4	CAMK2B, PRKACA, ADCY5, SPDYE16				
Signaling pathways regulating pluripotency of stem cells	16	WNT16, FGFR1, TCF7, ONECUT1, WNT4, FGFR4, WNT10A, WNT2B, WNT9A, NODAL, AKT3, GRB2, ACVR1C, JAK2, ESRRB, ISL1	Cellular community – eukaryotes	73		
JAK-STAT signaling pathway	19	IFNGR1, IL6, IL4R, CSF3, CSF2, IL2RG, IL20RB, IL23A, GFAP, IL3RA, AKT3, CNTFR, GRB2, PDGFRA, CDKN1A, JAK2, MPL, IL13RA2, FHL1	Signal transduction	231	Environmental information processing	327
MAPK signaling pathway	39	BDNF, EFNA2, FGFR1, CACNB2, ANGPT1, MAP2K6, FGFR4, PTPRR, RASGRP2, CACNA1C, KIT, JUN, EREG, FGF9, FGF3, IL1B, VEGFD, CACNA2D1, MEF2C, PRKCG, AKT3, GRB2, PRKACA, FGF18, CSF1R, PDGFRA, MAPK10, IL1A, PGF, RASGRP1, CACNB4, IGF2, CACNA1E, CACNA1G, HSPA6, CACNA1B, CACNA1D, FGF17, NGF				
FoxO signaling pathway	16	SLC2A4, IL6, TPTEP2-CSNK1E, PLK2, AGAP2, ATM, RAG2, SOD2, PCK2, PRKAG2, AKT3, GRB2, S1PR1, CDKN1A, MAPK10, PCK1				

Num: number.

## 4 Discussion

Gemcitabine combined with platinum-based chemotherapeutics is still the gold standard treatment for advanced GBC [3]. Several clinical trials of targeted therapy have been conducted [7,8] with the aim of enhancing overall survival. However, high molecular heterogeneity and acquired resistance are inevitable, often resulting in treatment failure. Therefore, identification of more potential targets in resistant cells is an effective strategy to combat these challenges, especially in cancer with a significantly lower incidence.

A gradual increase in drug concentration is a widely used method to establish resistant cells. Generally, the drug concentration at the site of cell death (20%) is helpful for primary induction; thus, 0.05 μM OXA, which induces 10–20% of cell death, was selected as the initial dose. After 5 months of induction, the two OXA-resistant GBC cells were completely adapted to 5 μM OXA. The parental GBC cell lines were slightly resistant to OXA, and the IC_50_ values were approximately 29 μM and 7 μM, which were much higher than those of other cancer cells [12,13]. This may be due to the intrinsic resistance of the cell line, which is consistent with the clinical results that advanced GBC is resistant to most chemotherapeutic agents [14].

The resistant cell lines showed decreased proliferation potency, similar to that shown in previous studies [13,15], and increased clone formation ability with the addition of 1 μM OXA. This indicated a strong tolerance to OXA, as well as a significantly higher stemness of the resistant cells, which was then verified using a soft agar colony formation assay. In addition, enhanced migration ability and the occurrence of EMT were also detected, although the reduction in E-cadherin was not significant in GBC-SD/OXA cells. Upregulation of certain transporters has been identified as an important mechanism for resistance to chemotherapeutic agents [16]. In addition to P-gp, other multidrug resistance-associated proteins (MRPs), such as MRP1 and MPR3, were also detected; however, their expression alterations were not consistent in the two resistant cell lines. Therefore, only the results of P-gp increased in the two cell lines were considered. The difference in the expression of the MRPs also reflected the heterogeneity of the two parental cells in the process of acquired resistance.

RNA-seq analysis indicated that the expression profiles of the two parental cell lines varied significantly. It has been reported that the genomic alterations of GBC are different from those of other biliary system cancers and also depend on ethnicity [17]. We found that the individual differences were also significant, although the parental cell lines were both isolated from Chinese patients of the same age. This may also explain why the responses varied significantly after patients with similar HER2 amplifications received the same targeting therapy. Under the threshold of |log2FC| > 1, a total of 4,675 DGs were screened in the two resistant cell lines. We also attempted to screen the DGs combined with the *p*-value (<0.05); however, only 23 genes were obtained under the filters |log2FC| > 1 and *p* < 0.05. Therefore, only the FC value was used as a screening condition. qPCR validation indicated that five genes (CSF2, S1PR1, DLX1, MED29, and CXCL8) were increased in the two resistant cell lines, accounting for 41.67% of the 12 randomly selected DGs. Therefore, nearly 1,948 DGs were verified and require further study.

The protein encoded by CSF2, also known as granulocyte macrophage colony-stimulating factor (GM-CSF), functions as a cytokine that stimulates the growth and differentiation of stem cells [18]. It has been reported that CSF2 is closely associated with the poor prognosis of some cancers [19,20] and is also an important signaling molecule in the regulation of cancer cell stemness [21]. Stem cells can also secrete CSF2 in response to various injuries [22]. Similarly, higher levels of S1PR1 were also found to be related to shorter overall survival, and overexpression of S1PR1 significantly promotes EMT, invasion, and stemness of cancer cells [23]. These insights indicate that the enhanced colony formation ability of resistant cells may be attributed to the upregulations of CSF2 and S1PR1, and additional assays are required to confirm this.

KEGG classification analysis is a useful tool for the rapid identification of the biological roles of DGs. The decreased proliferation potency, increased migration, and clone formation abilities of resistant cells were all traceable following DGs classification in relevant pathways. Other resistance-related pathways also provided new insights for re-examining chemoresistance. Targeting EGFR is one of the most common strategies in clinical oncology, and TKI combined with doublet chemotherapy result in a higher response rate; however, progression-free survival is not enhanced [24]. This may be due to activation of the EGFR TKI resistance pathway. Growth-arrest specific 6 (GAS6), a member of this pathway, is one of the major ligands of the AXL receptor. Increased levels of GAS6 can protect AXL from proteasome-mediated degradation, thereby resulting in resistance to EGFR inhibition [25]. As there is currently no inhibitor of GAS6, targeting AXL with its inhibitors brings a new chance to overcome TKI resistance. This is also helpful in enhancing the clinical effects of TKI combined with chemotherapy.

The upregulation of PD-L1 and p-ERK1/2 reflects the activation of these two pathways. PD-L1, expressed in the majority of tumor cells, is the ligand of PD-1 expressed in immune cells, and the activation of PD-1/PD-L1 results in the blockage of T-cell activation, thereby aiding tumor cells in evading immune surveillance [26,27]. ERK activation plays an important role in the development and progression of cancers [28]. To date, blocking the two targets with inhibitors such as JTX-4014 (anti-PD-L1) and MK-8353 (anti-p-ERK1/2) has shown some benefits in patients with advanced or refractory solid tumors [29,30], which may also be useful in advanced or resistant GBC patients. Generally, the activation of the ERK pathway leads to the enhanced proliferation of cancer cells; however, the proliferation of OXA-resistant cells was actually decreased compared to that of sensitive cells. The proliferation of cells is not only regulated by the ERK pathway; other pathways, such as the JAK/STAT3 and FoxO signaling pathways, in which many genes were dysregulated in OXA-resistant cells, also play important regulatory roles in cell proliferation [31,32]. Therefore, the detected slowed proliferation is a final neutralizing result of different activated/inactivated levels of various pathways.

One limitation of this study is that only two pathways were detected, and more drug-resistance-relevant pathways require detection and verification. Another limitation is that the role of inhibiting the verified pathways was not investigated, which would help reverse OXA-resistance.

In summary, the present study is the first to establish two OXA-resistant cell lines and determine the gene expression profile of the resistant cell lines. The validated genes, CSF2 and S1PR1, as well as the pathways of PD-L1 and ERK1/2, provide a foundation for the study of resistance mechanisms and the development of new targets. The invalidated genes in the expression profile also provide several potential targets, which are of great importance for the precise treatment of advanced or refractory GBC with extensive molecular heterogeneity.

## Supplementary Material

Supplementary Table
